# *Sulcisporasupratumida* sp. nov. (Phaeosphaeriaceae, Pleosporales) on *Anthoxanthumodoratum* from Italy

**DOI:** 10.3897/mycokeys.38.27729

**Published:** 2018-08-07

**Authors:** Indunil C. Senanayake, Rajesh Jeewon, Erio Camporesi, Kevin D. Hyde, Yu-Jia Zeng, Sheng-Li Tian, Ning Xie

**Affiliations:** 1 Shenzhen Key Laboratory of Microbial Genetic Engineering, College of Life Science and Oceanography, Shenzhen University, 3688, Nanhai Avenue, Nanshan, Shenzhen 518055, China Shenzhen University Shenzhen China; 2 Shenzhen Key Laboratory of Laser Engineering, College of Optoelectronic Engineering, Shenzhen University, Shenzhen 518060, China Mae Fah Luang University Chiang Rai Thailand; 3 Key Laboratory for Plant Biodiversity and Biogeography of East Asia (KLPB), Kunming Institute of Botany, Chinese Academy of Science, 132 Lanhei Road, Kunming 650201, Yunnan, China Kunming Institute of Botany, Chinese Academy of Science Kunming China; 4 Center of Excellence in Fungal Research, Mae Fah Luang University, Chiang Rai 57100, Thailand University of Mauritius Reduit Mauritius; 5 Department of Health Sciences, Faculty of Science, University of Mauritius, Reduit, 80837, Mauritius A.M.B. Gruppo Micologico Forlivese “Antonio Cicognani” Forlì Italy; 6 A.M.B. Gruppo Micologico Forlivese “Antonio Cicognani”, Via Roma 18, Forlì, Italy A.M.B. Circolo Micologico “Giovanni Carini” Brescia Italy; 7 A.M.B. Circolo Micologico “Giovanni Carini”, C.P. 314, Brescia, Italy Società per gli Studi Naturalisticidella Romagna Bagnacavallo Italy; 8 Società per gli Studi Naturalisticidella Romagna, C.P. 144, Bagnacavallo (RA), Italy Kunming Institute of Botany, Chinese Academy of Sciences Yunnan Thailand

**Keywords:** Combined gene analysis, Dothideomycetes, graminicolous fungi, new species, spore septation

## Abstract

*Sulcispora* is typified by *S.pleurospora*. We collected a sulcispora-like taxon on leaves of *Anthoxanthumodoratum* L. in Italy and obtained single ascospore isolates. Combined ITS, LSU, SSU and tef1 sequence analyses suggested that *Sulcispora* is placed in the family Phaeosphaeriaceae and a newly collected *Sulcispora* species is introduced here as *S.supratumida* sp. nov. Detailed descriptions and illustrations are provided for *Sulcisporasupratumida* and it is compared with the type species, *S.pleurospora*.

## Introduction

Phaeosphaeriaceae is a highly diverse and large family in the order Pleosporales (Hyde et al. 2013) with more than 42 accepted genera (Hyde et al. 2017; Karunarathna et al. 2017; Wanasinghe et al. 2018). Members of Phaeosphaeriaceae are pathogens or hyper-parasites on living plants and humans and saprobes of decaying plant matter (Tennakoon et al. 2016; Ahmed et al. 2017).

*Sulcispora* was proposed by Shoemaker and Babcock (1989) as a monotypic genus to accommodate *Sulcisporapleurospora* (≡ *Phaeosphaeriapleurospora* Niessl). Some morphological characters of *Phaeosphaeriapleurospora* did not fit within species concepts of *Phaeosphaeria* and Shoemaker and Babcock (1989), therefore, introduced the genus *Sulcispora*. The genus name refers to the numerous furrows on the ascospore wall (Shoemaker and Babcock 1989). *Sulcisporapleurospora* has been reported on monocotyledonous hosts in genera such as *Anthoxanthum*, *Carex*, *Deschampsia*, *Sesleria* and *Tofieldia* (Leuchtmann 1984; Shoemaker and Babcock 1989).

In this study, we collected sulcispora-like species associated with leaf spots of *Anthoxanthumodoratum* in Italy. We compared the morphological characters of our collection with the isotype of *Sulcisporapleurospora*. Morphologically, our collection differs from the type species of *Sulcispora*, *S.pleurospora*. Therefore, we introduce our collection as a new species. Combined ITS, LSU, SSU and tef1 sequence analysis including taxa in Phaeosphaeriaceae indicates that the here-studied fungus grouped with “*Phaeosphaeriapleurospora*” (CBS 460.84) with high support value.

## Methods

### Sample collection, specimen examination and single spore isolation

Specimens were collected from *Anthoxanthumodoratum* L. from Italy in 2013. They were examined and photographed using a Carl Zeiss Discovery V8 stereo-microscope fitted with Axiocam. Sections of ascomata were taken by hand under a stereo-microscope. Sections and other micro-morphological characters were photographed using a Nikon Eclipse 80i compound microscope fitted with a Canon 450D digital camera. All microscopic measurements were made with Tarosoft image framework (v. 0.9.0.7). Colony characteristics were recorded from cultures grown on Malt Extract Agar (MEA).

Single spore isolation was carried out following the method described by Chomnunti et al. (2014). Germinated ascospores were aseptically transferred into fresh MEA plates and incubated at 20 °C to obtain pure cultures and later transferred to MEA slants and stored at 4 °C for further study. The holotype and paratype specimens were deposited at the Mae Fah Luang University (MFLU) fungaria and the herbarium of Kunming Institute of Botany, Chinese Academy of Sciences (HKAS), respectively. Living cultures were deposited at the Mae Fah Luang Culture Collection (MFLUCC). MycoBank (http://www.mycobank.org/) and Facesoffungi (Jayasiri et al. 2015) numbers were obtained for the new strain. The new species was established based on recommendations outlined by Jeewon and Hyde (2016).

### DNA extraction, PCR amplification and DNA sequencing

Fresh fungal mycelium grown on MEA for four weeks at 20°C was used for DNA extraction (Jeewon et al. 2002). Genomic DNA extraction and PCR reactions were carried out using ITS4/ITS5 for internal transcribed spacer nrDNA (ITS), LR5/LROR for large subunit nrDNA (LSU), NS1/NS4 for large subunit nrDNA (SSU) and 983F/2218R for translation elongation factor 1 (tef1) genes according to the same protocol of Maharachchikumbura et al. (2012). The PCR products were observed on 1% agarose electrophoresis gel stained with ethidium bromide. Purification and sequencing of PCR products were carried out at the Kunming Institute of Botany, Chinese Academy of Science, Kunming, China. Sequence quality was checked and sequences were condensed with DNASTAR Lasergene v.7.1. Sequences derived in this study were deposited in GenBank (Table 1).

**Table 1.** Isolates used in this study and their GenBank and culture accession numbers. The strain of *Sulcisporasupratumida* sp. nov. is set in bold font and all ex-type strains are annotated with “^T^”.

Taxon	Culture accession no	ITS	LSU	SSU	tef-1
* Allophaeosphaeria muriformia *	MFLUCC 13-0349^T^	KP765680	KP765681	KP765682	–
* A. subcylindrospora *	MFLUCC 13-0380^T^	KT314184	KT314183	KT314185	–
* Amarenographium ammophilae *	MFLUCC 16-0296^T^	KU848196	KU848197	KU848198	MG520894
* Ampelomyces quisqualis *	CBS 129.79^T^	HQ108038	JX681064	EU754029	–
* Bhatiellae rosae *	MFLUCC 17-0664^T^	MG828873	MG828989	MG829101	–
* Chaetosphaeronema hispidulum *	CBS 216.75	KF251148	KF251652	EU754045	–
* Dactylidina dactylidis *	MFLUCC 14-0963^T^	MG828887	MG829003	MG829114	MG829199
* D. shoemakeri *	MFLUCC 14-0966^T^	MG828886	MG829002	MG829113	MG829200
* Dematiopleospora mariae *	MFLUCC 13-0612^T^	–	KJ749653	KJ749652	KJ749655
* Didymella exigua *	CBS 183.55^T^	GU237794	EU754155	EU754056	–
* Didymocyrtis caloplacae *	CBS 129338	JQ238641	JQ238643	–	–
* D. ficuzzae *	CBS 128019	KP170647	JQ238616	–	–
* D. cladoniicola *	CBS 128026	JQ238626	–	–	–
* Embarria clematidis *	MFLUCC 14-0976^T^	MG828871	MG828987	MG829099	MG829194
* Entodesmium rude *	CBS 650.86	–	GU301812	–	GU349012
* Equiseticola fusispora *	MFLUCC 14-0522^T^	KU987668	KU987669	KU987670	MG520895
* Galliicola pseudophaeosphaeria *	MFLUCC 14-0527^T^	KT326692	KT326693	–	MG829203
* Hawksworthiana clematidicola *	MFLUCC 14-0910^T^	MG828901	MG829011	MG829120	MG829202
* H. lonicerae *	MFLUCC 14-0955^T^	MG828902	MG829012	MG829121	MG829203
* Italica achilleae *	MFLUCC 14-0959^T^	MG828903	MG829013	MG829122	MG829204
* Juncaceicola alpine *	CBS 456.84	KF251181	KF251684	–	–
* J. luzulae *	MFLUCC 16-0780	KX449529	KX449530	KX449531	MG520898
* Leptospora rubella *	CPC 11006	DQ195780	DQ195792	DQ195803	–
* Loratospora aestuarii *	JK 5535B	–	GU301838	GU296168	–
* L. luzulae *	MFLUCC 14-0826	KT328497	KT328495	KT328496	–
* Melnikia anthoxanthii *	MFLUCC 14-1010^T^	KU848205	KU848204	–	–
* Muriphaeosphaeria galatellae *	MFLUCC 14-0614^T^	KT438333	KT438329	KT438331	MG520900
* Neosetophoma italica *	MFLUCC14-0826^T^	KP711356	KP711361	KP711366	–
* N. samarorum *	CBS 138.96^T^	FJ427061	KF251664	GQ387517	–
* Neostagonospora caricis *	CBS 135092/S616^T^	KF251163	KF251667	–	–
* N. eligiae *	CBS 135101^T^	KF251164	KF251668	–	–
* Nodulosphaeria hirta *	MFLUCC 13-0867	KU708849	KU708845	KU708841	KU708853
* N. senecionis *	MFLUCC 15-1297	KT290257	KT290258	KT290259	–
* Ophiobolus cirsii *	MFLUCC 13-0218^T^	KM014664	KM014662	KM014663	–
* O. disseminans *	AS2L14-6	–	–	KP117305	–
* Ophiosphaerella agrostidis *	MFLUCC 11-0152^T^	KM434271	KM434281	KM434290	KM434299
* Paraleptosphaeria dryadis *	CBS 643.86	J F740213	GU301828	KC584632	GU349009
* Paraphoma chrysanthemicola *	CBS 522.66	FJ426985	KF251670	GQ387521	–
* P. radicina *	CBS 111.79^T^	KF251172	KF251676	EU754092	–
* Parastagonospora nodorum *	CBS 110109^T^	KF251177	KF251681	EU754076	–
* P. poagena *	CBS 136776^T^	KJ869116	KJ869174	–	–
* Phaeosphaeria chiangraina *	MFLUCC 13-0231^T^	KM434270	KM434280	KM434289	KM434298
* P. oryzae *	CBS 110110^T^	KF251186	KF251689	GQ387530	–
* P. papayae *	S528	KF251187	KF251690	–	–
* Phaeosphaeria pleurospora *	CBS 460.84	AF439498	–	–	–
* Phaeosphaeriopsis glaucopunnctata *	MFLUCC 13-0265^T^	KJ522473	KJ522477	KJ522481	MG520918
* P. triseptata *	MFLUCC 13-0271^T^	KJ522475	KJ522479	KJ522484	MG520919
* Poaceicola arundinis *	MFLUCC 15-0702^T^	KU058716	KU058726	–	MG520921
* P. italica *	MFLUCC 13-0267^T^	KX926421	KX910094	KX950409	MG520924
* Populocrescntia forlicesesensis *	MFLU 15-0651^T^	KT306948	KT306952	KT306955	MG520925
* Premilcurensis senecionis *	MFLUCC 13-0575^T^	KT728365	KT728366	–	–
*Sclerostagonospora* sp.	CBS 123538	FJ372393	FJ372410	–	–
* Scolicosporium minkeviciusii *	MFLUCC 12-0089^T^	–	KF366382	KF366383	–
* Septoriella leuchtmannii *	CBS 459.84^T^	KF251188	KF251691	–	–
* Setomelanomma holmii *	CBS 110217	–	GU301871	GQ387572	GU349028
* Setophoma sacchari *	CBS 333.39^T^	KF251245	KF251748	GQ387525	–
* S. terrestris *	CBS 335.29^T^	KF251246	KF251749	GQ387526	–
*** Sulcispora supratumida ***	**MFLUCC 14-0995**	**KP271443**	**KP271444**	**KP271445**	**MH665366**
* Tintelnotia destructans *	CBS 127737^T^	NR_147684	NG_058274	KY090698	–
* T. destructans *	CBS 137534	–	KY090663	KY090697	–
* Vagicola chlamydospora *	MFLUCC 15-0177^T^	KU163658	KU163654	–	–
* V. vagans *	CBS 604.86	KF251193	KF251696	–	–
* Vrystaatia aloeicola *	CBS 135107	KF251278	KF251781	–	–
* Wojnowicia dactylidis *	MFLUCC 13-0735^T^	KP744470	KP684149	KP684150	–
* W. lonicerae *	MFLUCC 13-0737^T^	KP744471	KP684151	KP684152	–
* Wojnowiciella eucalypti *	CPC 25024^T^	KR476741	KR476774	–	LT990617
* Xenoseptoria neosaccardoi *	CBS 128665^T^	KF251281	KF251784	–	–
* X. neosaccardoi *	CBS 120.43	KF251280	KF251783	–	–
* Yunnanensis phragmitis *	MFLUCC 17-0315^T^	MF684862	MF684863	MF684867	MF683624
* Y. phragmitis *	MFLUCC 17-1365^T^	MF684869	MF684865	MF684864	MF683625

**CBS**: Westerdijk Fungal Biodiversity Institute, Utrecht, The Netherlands; **CPC**: Culture collection of Pedro Crous, housed at CBS-KNAW; **MFLUCC**: Mae Fah Luang University Culture Collection, Chiang Rai, Thailand.

### Sequence alignment and phylogenetic analysis

BLASTn searches were made using the newly generated sequences to assist in taxon sampling for phylogenetic analyses. In addition, representatives of the Phaeosphaeriaceae were selected following Tennakoon et al. (2016) and Wanasinghe et al. (2018) (Table 1). Combined multi-locus sequence data of ITS, LSU, SSU and tef1 regions were aligned using default settings of MAFFT v.7 (Katoh et al. 2017) and manually adjusted using BioEdit 7.1.3 (Hall 1999) to allow maximum alignment and minimum gaps. Maximum likelihood analysis was performed by RAxML (Stamatakis and Alachiotis 2010) implemented in raxmlGUIv.1.3 (Silvestro and Michalak 2012). The search strategy was set to rapid bootstrapping and the analysis carried out using the GTRGAMMAI model of nucleotide substitution with 1000 replicates. The model of evolution was estimated by using MrModeltest 2.2 (Nylander 2004).

For the Bayesian inference (BI) analyses of the individual loci and concatenated ITS, LSU, SSU and tef-1 alignment, the above mentioned model test was used to determine the best fitting nucleotide substitution model settings for MrBayes v. 3.0b4. A dirichlet state frequency was predicted for all three data partitions and GTR+I+G as the best model for all single gene and combined datasets. The heating parameter was set to 0.2 and trees were saved every 1000 generations (Ronquist and Huelsenbeck 2003). The Markov Chain Monte Carlo (MCMC) analysis of four chains started in parallel from a random tree topology. The Bayesian analysis lasted 10,000,000 generations (average standard deviation of split frequencies value = 0.0098) and the consensus trees and posterior probabilities were calculated from the 9,998,000 trees sampled after discarding the first 20% of generations as burn-in. Trees obtained in this study were deposited in TreeBASE under accession number S22938. The phylogram was visualised in FigTree v. 1.2.2 (Rambaut and Drummond 2008).

## Results

### Phylogenetic inferences

**Figure 1.**
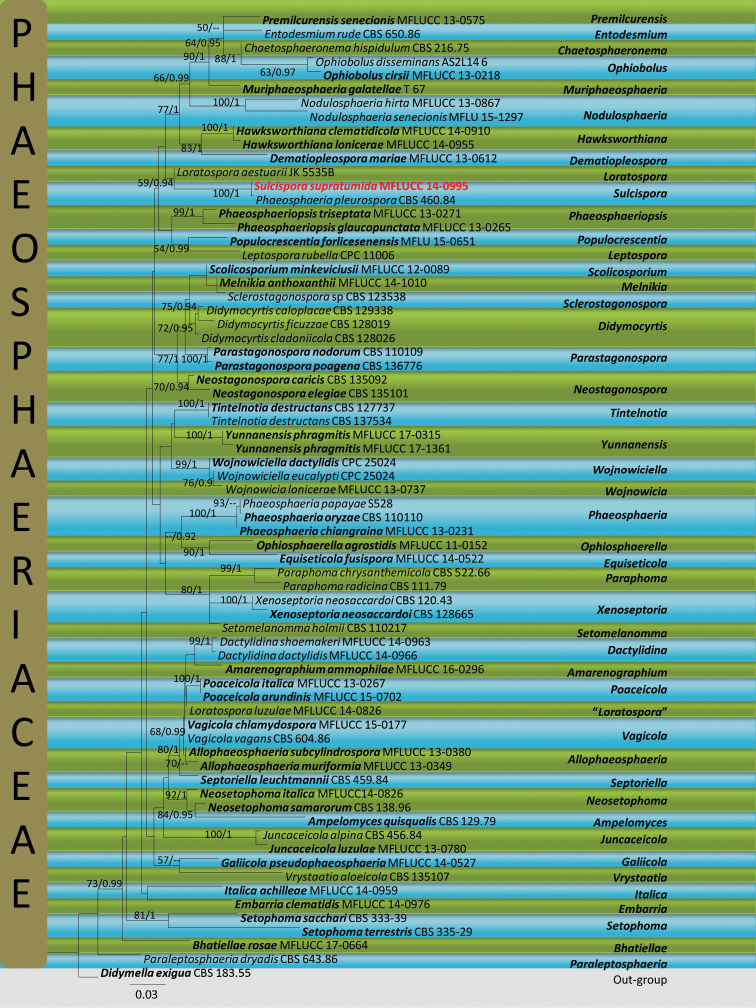
Maximum likelihood majority rule consensus tree based on a combined dataset of ITS, LSU, SSU and tef-1 sequences. Bootstrap support values ≥50% and Bayesian inference (BI) ≥0.9 are given at the nodes. The tree is rooted to *Didymellaexigua* (CBS 183.55). The culture accession numbers are given after the species names. All ex-type strains are in bold. The newly introduced species from this study is in bold red.

The combined ITS, LSU, SSU and tef-1 sequence data set comprised 69 strains of Phaeosphaeriaceae with *Didymellaexigua* as the outgroup taxon. All individual trees generated under different criteria and from single gene datasets were essentially similar in topology and not significantly different from the tree generated from the concatenated dataset. Maximum likelihood analysis with 1000 bootstrap replicates yielded a tree with the likelihood value of ln: -13019.593920 and the following model parameters: alpha: 0.144187; Π(A): 0.245356, Π(C): 0.229408, Π(G): 0.267562 and Π(T): 0.257674. The best scoring RAxML tree is shown in Figure 1. Maximum likelihood bootstrap values ≥50% and Bayesian inference (BI) ≥0.9 are given at each node.

The phylogenetic trees obtained from maximum likelihood were topologically congruent to previous studies on Phaeosphaeriaceae (Phookamsak et al. 2014; Thambugala et al. 2014; Tennakoon et al. 2016; Karunarathna et al. 2017; Wanasinghe et al. 2018). This phylogenetic analysis showed the placement of 45 genera within Phaeosphaeriaceae. The here-studied strain clustered with CBS 460.84 (one of Leuchtmann’s Swiss strains of *S.pleurospora* from *Carexfirma*) with 100% bootstrap support value. The ITS sequence of the CBS 460.84 is almost identical to our strain (MFLUCC 14–0995). However no LSU, SSU and tef-1 sequences were obtained from CBS 460.84 in GenBank. The herbarium specimen of CBS 460.84 is in Westerdijk Fungal Biodiversity Institute (CBS) under accession number CBS H-15991 (SWITZERLAND, Kt. Graubünden, Zügenschlucht near Davos, *Carexfirma*, A. Leuchtmann). However, CBS has presently stopped sending specimens on loan, hence we could not compare morphological characters of the here studied strain with CBS 460.84. Additionally *Sulcispora* sisterly clustered with the type species of *Loratospora*, *L.aestuarii* with low support and the second species of *Loratospora*, *L.luzulae*. was distantly clustered.

### Taxonomy

#### Sulcispora supratumida

Taxon classificationFungiPleosporalesPleosporaceae

Senan., Camporesi & K.D. Hyde sp. nov.

826887

Figure 2

##### Etymology.

The species epithet is based on the two Latin words “supra” meaning upper and “tumidus” meaning swollen, referring to the position of swollen cells of ascospores.

##### Type.

ITALY. Province of Forli-Cesena, Premilcuore, Passodella Valbura, on dead leaves of *Anthoxanthumodoratum* L. (Poaceae), 25 May 2013, Erio Camporesi, IT 1306 (MFLU 15–0038, holotype; HKAS 83865, paratype): living cultures, MFLUCC 14–0995.

##### Description.

*Saprobic* on leaves of *Anthoxanthumodoratum* L., visible as black spots, occurring on the upper surface of entire leaf. *Sexual morph*. *Ascomata* 110–150 × 90–140 µm (x– = 140–125 µm, n = 10), scattered, solitary, immersed, uniloculate, globose, black. *Ostiole* 35–40 µm (x– = 39 µm, n = 10) wide, papillate, central, periphysate. *Periphyses* 15–20 µm long, hyaline. *Peridium* comprising 2–4 layers of brown to dark brown, thick-walled, cells of *textura angularis* to *textura globularis*. *Hamathecium* comprising 2–4 µm wide, cellular, hyaline, branched, septate, pseudoparaphyses, constricted at the septa, anastomosing mostly above the asci and embedded in a mucilaginous matrix. *Asci* 85–125 × 20–35 µm (x– = 100 × 30 µm, n = 20), 8-spored, few, bitunicate, fissitunicate, subglobose to clavate, short pedicellate, apically rounded, with an ocular chamber, arising from the base of the ascoma and attached to parenchymatous cell matrix at base. *Ascospores* 30–35 × 6–9 µm (x– = 35 × 7 µm, n = 25), bi-seriate to tri-seriate, narrowly fusiform, narrowing towards the end cells, reddish to dark brown, 6-septate, second septum supra-median, slightly constricted, not constricted at other septa, second segment swollen, straight, with 12–16 longitudinal furrows on surface, lacking a mucilaginous sheath. *Asexualmorph*. Undetermined.

**Figure 2.**
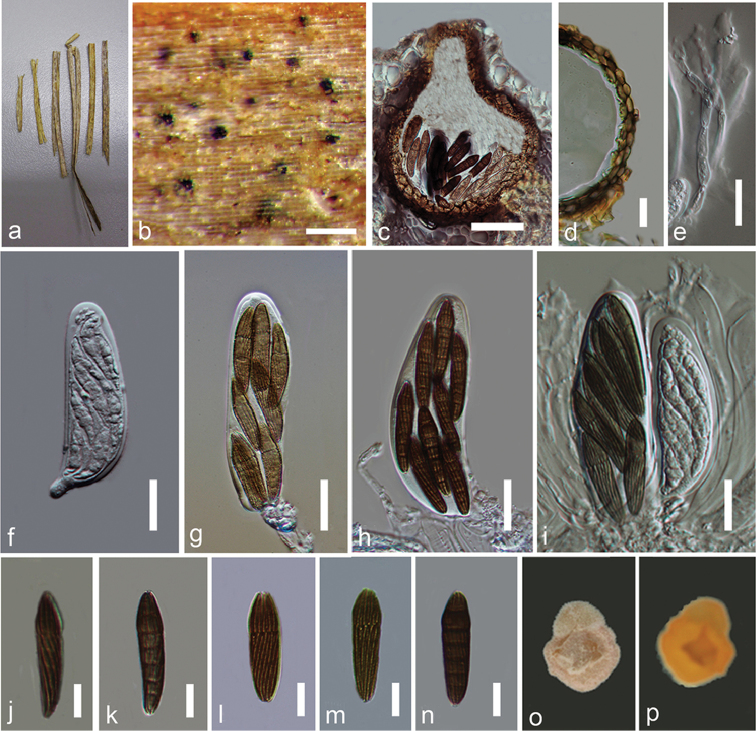
*Sulcisporasupratumida* (MFLU 15–0038). **a** Leaves of *Anthoxanthumodoratum***b** Appearance of ascomata on host surface **c** Cross section of ascoma **d** Peridium **e** Pseudoparaphyses **f–i** Asci **j–n**Ascospores**o** Upper surface of the culture **p** Lower surface of the culture. Scale bars: 200 µm (**b**), 50 µm (**c**), 20 µm (**d–i**), 10 µm (**j–n**).

##### Culture characteristics.

2 cm diameter after 4 weeks incubated in dark at 25 °C on MEA, pinkish-white, circular, slightly woolly, margin lobate, effuse, lacking aerial mycelium, tightly attached to the media.

## Discussion

Shoemaker and Babcock (1989) observed type specimens of *Phaeosphaeriapleurospora* and found that the ascospores of *P.pleurospora* with striated ornamented walls are different to those of other genera in Phaeosphaeriaceae. Hence, they introduced the genus *Sulcispora* to accommodate *P.pleurospora* and placed it in Phaeosphaeriaceae. *Sulcisporapleurospora* has some similarities with *Phaeosphaeriaexarata* Shoemaker & C.E. Babc., in having very large cells in the peridium, ascospores with a continuous sheath and ornamented wall of ascospores with coarse, longitudinal ridges (Shoemaker and Babcock 1989).

In this study, a combined gene sequence analysis of taxa amongst the Phaeosphaeriaceae provides substantial evidence to support *Sulcispora* as a distinct genus in Phaeosphaeriaceae. *Sulcispora* differs from other genera in having immersed ascomata with a relatively thin wall, cellular pseudoparaphyses, short pedicellate asci and brown ascospores (Phookamsak et al. 2014).

Leuchtmann (1984) reported variation of ascospore septation amongst several collections of *Phaeosphaeriapleurospora* from different host plants. *Phaeosphaeriapleurospora*, collected from *Sesleriacaerulea* (L.) Ard. and *Carexfirma* Mygind ex Host, usually formed 6-septate ascospores and the second segment was swollen. Our collection is morphologically identical to Leuchtmann’s collection. However, the isotype and some of Leuchtmann’s collections from other host plants had 5–8-septate ascospores and the third or fourth segment was swollen (Table 2). Therefore Leuchtmann (1984) characterised *Phaeosphaeriapleurospora* as a species with 5–8 septate ascospores. However, Leuchtmann’s collection of *Sulcisporapleurospora* is likely to comprise more than a single species and possibly constitutes a species complex.

**Table 2.** Ascospore morphology comparison of *Sulcispora* species

Species name	Herbarium type data	Host	No of septa	Swollen cell	Reference
* Sulcispora pleurospora *	FH 196419 (isotype)	*Deschampsiacespitosa* (Poaceae)	5–6	3^rd^	Shoemaker and Babcock 1989
	F6952, F6949, F6951 (isotype)	*Deschampsiacespitosa* (Poaceae)	6	3^rd^	In this study
	M (1 collection), ZT (8 collections)	6 monocotyledonous hosts,1 dicotyledonous host	6–8	3^rd^ or 4^th^	Leuchtmann 1984
* Sulcispora supratumida *	ZT (6 collections)	*Seleriacaerulea* (Poaceae)*Carexfirma* (Cyperaceae)	6	2^nd^	Leuchtmann 1984
	MFLU 15-0038 (holotype)	*Anthoxanthumodoratum* (Poaceae)	6	2^nd^	In this study

Based on the morphology, we identified our collection as different from the isotype of *Sulcisporapleurospora*. Hence, we introduced a new species as *Sulcisporasupratumida* sp. nov. However, the ITS sequence of our strain clustered with that of CBS 460.84 (one of Leuchtmann’s Swiss strain of *S.pleurospora* from *Carexfirma*) with 100% bootstrap support value. There are only two base pair differences between the ITS regions of both strains. Since there are no sequence data of other DNA regions of *Sulcisporapleurospora* deposited in GenBank, we could not confirm whether or not CBS 460.84 is *Sulcisporasupratumida*. However, it would eventually be practical to obtain the living strain of CBS 460.84 and generate further sequence data.

### Keys for species in *Sulcispora*

**Table:** 

1	Ascomata erumpent, long papillate, 5–8-septated, ascospores with 3^rd^ swollen cell	*** S. pleurospora ***
–	Ascomata immersed, short papillate, 6-septated, ascospores with 2^nd^ swollen cell	*** S. supratumida ***

## Supplementary Material

XML Treatment for Sulcispora supratumida
